# Epidemiological Characteristics of Hemorrhagic Fever with Renal Syndrome in Longyou County, China

**DOI:** 10.3390/v17030313

**Published:** 2025-02-25

**Authors:** Jing Ni, Dejun Kong, Zhongbing Chen, Weiming Zeng, Bingdong Zhan, Zhenyu Gong

**Affiliations:** 1 School of Public Health, Hangzhou Medical College, Hangzhou 310013, China; 130232023444@hmc.edu.cn; 2 Department of Communicable Disease Control and Prevention, Zhejiang Provincial Center for Disease Control and Prevention, Hangzhou 310051, China; 3 Longyou Centre for Disease Control and Prevention, Quzhou 324400, China; 13676603525@139.com (D.K.); chenzhongbing9@163.com (Z.C.); zengweiming55321@sina.com (W.Z.); 4 Quzhou Centre for Disease Control and Prevention, Quzhou 324000, China; 5 Zhejiang Key Lab of Vaccine, Infectious Disease Prevention and Control, Hangzhou 310051, China

**Keywords:** HFRS, epidemiologic features, surveillance, rodent, integrated monitoring

## Abstract

(1) Background: We aimed to analyze the epidemiological characteristics of hemorrhagic fever with renal syndrome (HFRS) in Longyou County and to provide a basis for the future response to this disease. (2) Methods: Data on hemorrhagic fever and host animals were collected from 2011 to 2023. Descriptive methods were used to analyze the epidemic. The R4.4.1 software was used to show how the host density relates to the virus levels, temperature, and rainfall and to predict the host density. (3) Results: We observed 58 cases of hemorrhagic fever, the majority of which occurred in farmers. There were two incidence peaks each year during the spring and winter seasons, accounting for 22.41% and 43.10% of the total cases, respectively. The outdoor rodent population density was significantly and positively correlated with the outdoor rodent virus prevalence (R^2^ = 0.9411), serving as a robust predictor of the outdoor rodent virus prevalence. Additionally, the density of outdoor rodents exhibited a strong nonlinear relationship with the temperature and precipitation. (4) Conclusions: After hemorrhagic fever vaccination, rodent population density control, and rodent carrier rodent control from 1995 to 2000, the hemorrhagic fever epidemic was generally stable, and the epidemiological characteristics remained stable. In the future, we should continue to take active and effective comprehensive measures to intervene, further realize the effective control of HFRS, and prevent the recurrence of hemorrhagic fever epidemics.

## 1. Introduction

Hemorrhagic fever with renal syndrome (HFRS) is a viral disease transmitted to humans through contact with infected rodents and exposure to contaminated aerosols, posing a threat to global public health [1]. The causative agents of HFRS include Hantaan virus (HTNV), Seoul virus (SEOV), Pumara virus (PUUV), and other viruses of the genus Orthohanta, each of which causes disease with varying severity [2]. The main manifestations of hemorrhagic fever with renal syndrome are acute kidney injury, increased vascular permeability, and abnormal coagulation function [3]. The disease is widespread in several countries around the world, with large variability in the local prevalence rates, ranging from 1% to 5%, mainly in Asia and Europe [4,5]. China accounts for 90% of all HFRS cases globally. *Apodemus agrarius* (Pallas, 1771) and *Rattus norvegicus* (Berkenhout, 1769) are the main hosts and sources of infection for epidemic hemorrhagic fever in China [6]. Both reservoir animals are asymptomatic after infection, because hantaviruses have developed different escape mechanisms against the innate immune system during their long-term co-evolution within these host species [7]. The incidence of HFRS is closely related to the rodent population density, virulence rate, human activity, and climatic factors [8,9]. A recent study showed that the recurrence of HFRS occurs due to variations in the circulating strains [2]. Between 2004 and 2019, a total of 209,209 HFRS cases and 1855 deaths were reported in China. Chinese law classifies HFRS as a Class B infectious disease and requires the timely reporting of confirmed cases [9]. As one of the key HFRS-affected areas in China, Zhejiang Province has exhibited long-term efforts in the prevention and control of HFRS [10].

Before the 1990s, Zhejiang Province was a major area with a high incidence of HFRS in the country, reaching its peak in 1986. From 1991 to 2020, a total of 45,635 cases of HFRS and 287 deaths were reported in Zhejiang Province. After the first case of hemorrhagic fever with renal syndrome (HFRS) was reported in Longyou County in 1968, the epidemic continued to expand, and it became one of the high-incidence areas of HFRS in Zhejiang Province [11]. In 1995, Longyou County responded to the national call for large-scale rodent eradication and HFRS vaccination. Since 2000, the vaccination of key populations, the gradual improvement in living conditions in rural areas, and the enhancement of residents’ health knowledge have contributed to maintaining a relatively low incidence of HFRS. By 2010, the local HFRS incidence had been controlled at a stable level [12,13]. This study hypothesizes that the outdoor rodent density correlates with the outdoor rodent virus prevalence and serves as a robust predictor of outdoor rodent virus prevalence. The aim is to investigate the epidemiological features of HFRS in Longyou County, Zhejiang Province, from 2011 to 2023; elucidate the link between the rodent population density and disease incidence; and assess the influence of climatic variables (temperature and precipitation) on rodent dynamics and disease spread, thereby informing evidence-based HFRS control strategies.

## 2. Materials and Methods

### 2.1. Study Area

**Figure 1:**
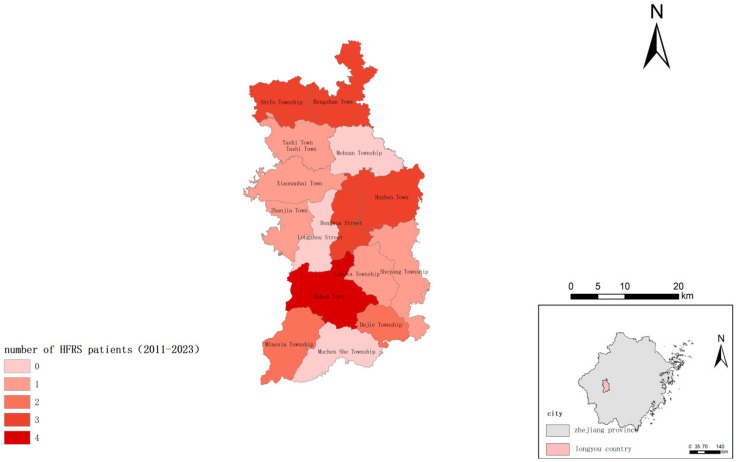
HFRS cases in Longyou County, 2011–2023.

Longyou County (Figure 1), located at 28°44′–29°17′ N, 119°02′-119°20′ E in the western hilly area of Quzhou City, Zhejiang Province, has a subtropical monsoon climate with mild temperatures, abundant rainfall, and distinct seasons. Its average annual temperature is 17.1 °C, with the temperature extremes ranging from −5.0 °C to 30.5 °C. The average annual precipitation is 1602.6 mm, and the average annual relative humidity is 79%. The county has a forest coverage rate of 54.79% and a permanent population of 360,200 and consists of 4 counties and 11 townships. These conditions provide a suitable environment for rodent reproduction, making Longyou County one of the high-HFRS areas in Zhejiang Province.

### 2.2. Data Collection

During data collection, we categorized HFRS cases into suspected cases, clinically diagnosed cases, and confirmed cases according to the diagnostic criteria of the health industry standards of the People’s Republic of China.

(1) Patients with a history of epidemiology and clinical manifestations, defined as suspected cases, are those who seek medical attention for fever or gastrointestinal symptoms;
(2) Cases suspected to be diagnosed with hypotension, renal function impairment, elevated peripheral blood cell counts, thrombocytopenia, and positive urine protein are defined as clinically diagnosed cases;
(3) Cases that meet one or more of the following clinical diagnostic criteria are defined as confirmed cases: positive serum-specific IgM antibodies; specific IgG antibodies that are four times higher than in the acute phase.

We collected daily meteorological data from the China Meteorological Data Sharing Service System (http://data.cma.cn/ (accessed on 1 November 2024)). Data were obtained from the Zhejiang Provincial Center for Disease Control and Prevention for 2011 to 2023, including the monthly case numbers in Longyou County and the demographics (age, gender, occupation, living area) of HFRS patients. We collected serum from the population and detected HFRS antibodies using immunofluorescence. The researchers involved in testing passed the training and examinations required to undertake testing at the Zhejiang Key Lab of Vaccines, Infectious Disease Prevention, and Control.

### 2.3. Rodent Surveillance

Rodent monitoring was conducted in a key town (Miaoxia Township), key rural area (Miaoxia Village), and key industries, utilizing the mouse trap method for monitoring. The monitoring periods included January, March, May, July, September, and November. Mouse traps were set up at these three locations to estimate the proportions of different rodent species and their activity levels. During the monitoring, 50 mouse traps were continuously set, using peanuts as bait. The traps were set at 4:00 PM on the previous day and were collected and checked at 6:00 AM on the following morning. Table 1 details the number of individuals captured each year and the species composition. We used direct immunofluorescence to detect HFRS-specific antigens in the rodent lungs and collected rodent blood to detect HFRS antibodies using the ELISA method. We calculated the rodent population density, positive cases (PA), positivity rate (PR), and rodent virus prevalence to assess the degree of HFRS prevalence among rodents. 

### 2.4. Statistical Methods

A descriptive analysis was conducted on the cases of hemorrhagic fever with renal syndrome, as well as the indoor and outdoor rodent population density and the differences in incidence between genders. Previous studies have shown that the annual HFRS carrying rate and rodent population density are linear, so a multiple linear regression (LR) analysis framework was used to quantitatively evaluate the combined effect of the outdoor rodent population density and outdoor rodent virus prevalence. The statistical model was based on the principle of least squares, aiming to optimize parameter estimation to accurately capture the linear dynamics between the independent and dependent variables. The mathematical representation of the model is as follows:

*Y* = *β*_0_ + *β*_1_*X* + *ϵ*

Among them, *Y* is the dependent variable, which is the outdoor rodent virus prevalence. *β*_0_ is the intercept, with an estimated value of 2.05. *β*_1_ is the regression coefficient of the independent variable (outdoor rodent population density), with an estimated value of 0.690. *X* is the value of the independent variable, which is the outdoor rodent population density. *ϵ* is the error term. To verify the applicability and interpretability of the model, this study conducted hypothesis testing on the significance of the regression coefficients and calculated the coefficient of determination (R-squared) to assess the model’s explanatory power regarding the variation in the outdoor toxicity index. In addition, by analyzing the residuals of the model, the normality and homoscedasticity of the error terms were assessed, ensuring the robustness of the model estimates.

To investigate the relationship between the temperature, precipitation, and outdoor rodent population density, this study employed a generalized additive model (GAM) [14]. The GAM was used to analyze the effects of the annual average temperature and annual average precipitation in Longyou County from 2011 to 2023 on the outdoor rodent population density and to predict the rodent population density under varying climatic conditions. The GAM framework is particularly suitable for capturing nonlinear relationships and interactions between variables. In the model, the temperature and precipitation were incorporated as smoothing terms, and their interaction effects were also considered. The model formula is expressed as

*Yi* = *β*0 + *f*1(*Ti*) + *f*2(*Pi*) + *f*3(*Ti*,*Pi*) + *ϵi*

Among them, *Yi* is the outdoor rodent population density of the *i*-th observation; *β*0 is the intercept term; *Ti* is the temperature of the *i*-th observation; *Pi* is the precipitation of the *i*-th observation; *f*1(*Ti*) is the smoothing function of the temperature, representing the nonlinear effect of the temperature on the outdoor rodent population density; *f2(Pi)* is the smoothing function of precipitation, representing the nonlinear effect of precipitation on the outdoor rodent density; *f*3(*Ti*,*Pi*) is the interaction smoothing function of the temperature and precipitation, representing the nonlinear effect of the temperature and precipitation together on the outdoor rodent density; and *ϵi* is the error term of the *i*-th observation, which is usually assumed to be independent and identically distributed, with a mean of 0 and constant variance. The model fitting utilized the restricted maximum likelihood (REML) method. Diagnostic plots were generated to assess the model’s fit, including the examination of residuals and smooth terms to ensure the robustness of the results.

The ggalluvial package in R4.4.1 was used to plot the age and gender distribution of HFRS in Longyou County. The ggplot2 (version 3.5.1) package was utilized to illustrate the indoor and outdoor rodent population density distribution; the lavaan (version 0.6.19) package was employed to visualize the relationship between the outdoor rodent population density and outdoor rodent virus prevalence; and the mgcv (version 1.9.1) package was used to visualize the impact of the temperature and precipitation on the outdoor rodent population density.

## 3. Result

**Figure 2:**
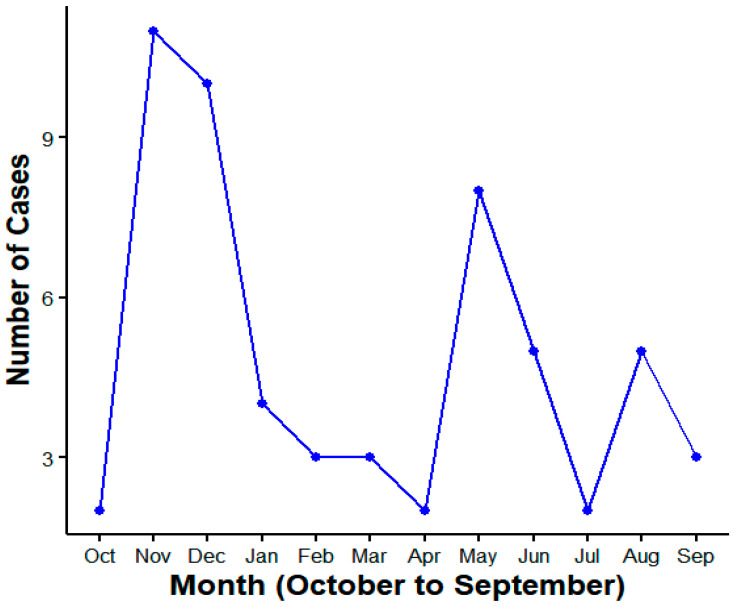
HFRS cases reported in Longyou County from 2011 to 2023.

From 2011 to 2023, Longyou County reported a total of 58 cases of HFRS, with an average annual incidence rate of 1.23 per 100,000. The annual incidence rate ranged from 0 to 3.31 per 100,000, indicating a generally low incidence rate; no deaths were reported. Cases were reported in all 15 towns (streets) of Longyou County, with the incidence rate in the southern mountainous towns being higher than that in the northern hilly towns, while the incidence rate in the central plain towns was the lowest. The epidemic showed occurrences throughout the year, with significant seasonality, featuring two peaks each year: the spring–summer peak in May to June accounted for 22.41% (13/58) of the cases, and the winter peak in November to January accounted for 43.10% (25/58) of the reported cases (Figure 2).

**Figure 3:**
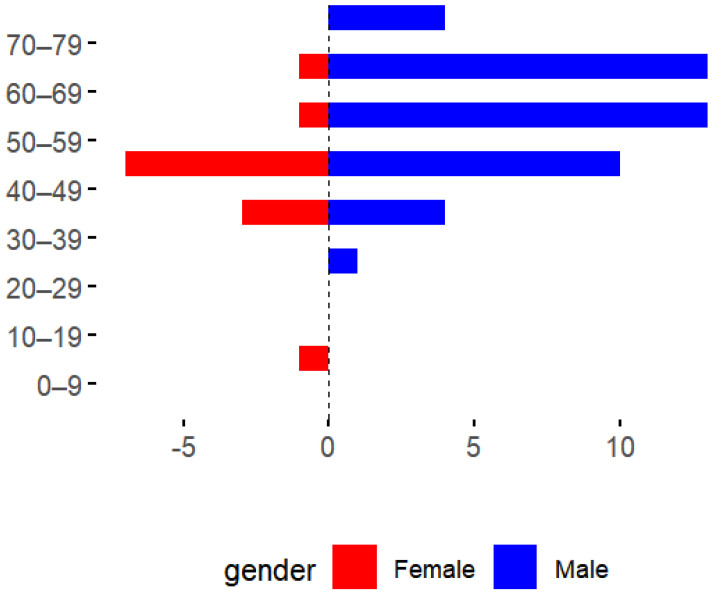
Age distribution of hemorrhagic fever cases in Longyou County, 2011–2023.

**Figure 4:**
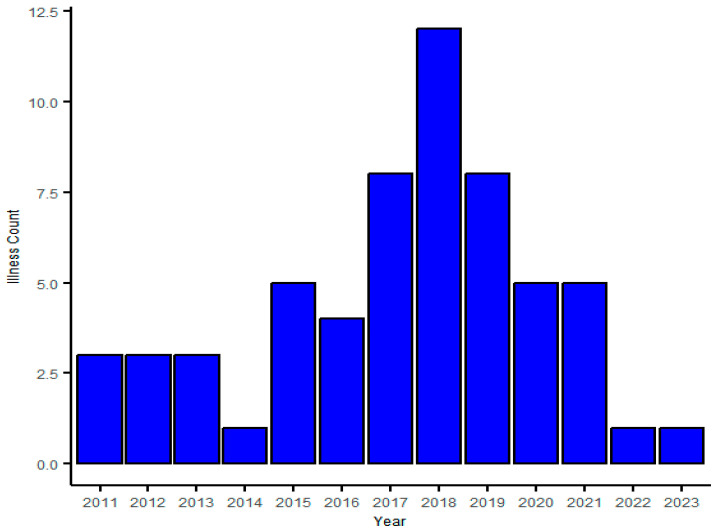
The annual number of HFRS cases in Longyou County, 2011–2023.

Among the 58 HFRS cases, 45 were males and 13 were females, with a sex ratio of 3.46:1. The youngest age at onset was 13 years old, the oldest was 72 years old, and most cases were reported in those aged 40–69 years old, accounting for 81.03% (47/58) of the total number of cases. Farmers accounted for 75.86% (44/58), followed by workers (8.62% (5/58)) (Figure 3 and Figure 4).

**Figure 5:**
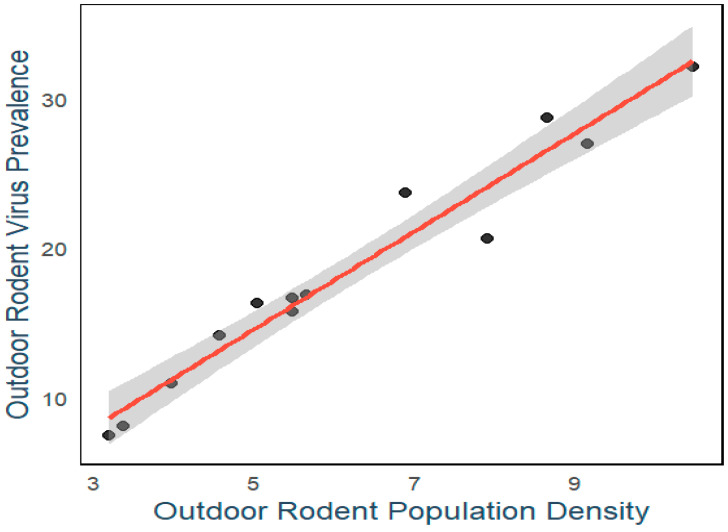
Correlation between outdoor rodent virus prevalence and outdoor rodent population density in Longyou County, 2011–2023.

The R^2^ of the linear regression model is 0.9411, indicating that the outdoor rodent population density can explain 94.11% of the variability in the outdoor rodent virus prevalence. This high R^2^ suggests that the model fits very well, and the outdoor rodent population density is a strong predictor of the outdoor rodent virus prevalence (Figure 5). The detailed statistical results of the regression model indicate that the estimated value of the intercept is 2.05 (*p* = 8.51 × 10^−8^), and the regression coefficient for the outdoor rodent population density is 0.690 (*p* = 1.21 × 10^−15^). This implies that, holding other variables constant, for each unit increase in the outdoor rodent population density, the outdoor rodent virus prevalence increases, on average, by 0.690 units. The significance test of the regression coefficient shows that the impact of the outdoor rodent population density on the outdoor rodent virus prevalence is significant.

In the 13 years from 2010 to 2022, a total of 26,991 traps were placed and 1446 rodents were captured, with an average rodent population density of 5.37%. The average indoor rodent population density was 40%, while the outdoor rodent population density was 5.42%. In the indoor environment, *Rattus tanezumi* was the main rodent species, accounting for 66.67%. In the wild, *Apodemus agrarius* was the main rodent species, accounting for 79.87%.

Among the 1446 rodent lung tissue samples examined, 42 (2.90%) tested positive for HFRS antigens. Among these, the positivity rate for lung tissue antigens of *Apodemus agrarius* (Table 1) captured outdoors was 3.33%; for *Rattus norvegicus*, it was 6.82%; for *Niviventer Confucius*, it was 2%; and for *Niniventer fulvescens*, it was 2.04%. No positive results were detected in the lung tissue of *Rattus tanezumi*, *Rattus norvegicus*, and other rodents captured indoors. In the analysis of the blood samples from the 1446 captured rodents, 103 samples tested positive for HFRS antibodies, yielding a positivity rate of 7.11%. Out of the 58 blood samples from indoor rodents, one was positive, resulting in a positivity rate of 1.72%, with *Rattus norvegicus* showing a positivity rate of 9.09%. Among the 1391 blood samples from outdoor rodents, 102 were positive, leading to a positivity rate of 7.33%, with *Apodemus agrarius* showing a positivity rate of 8.28%, *Rattus norvegicus* at 6.82%, *Niviventer confucianus* at 4%, *Rattus tanezumi* at 1.61%, *Niniventer fulvescens* at 8.16%, and other rodents at 0%. In 2011 and 2014, serum samples from 100 individuals who had been vaccinated against HFRS in 1995 were tested for HFRS antibodies, resulting in a total of 60 positive cases, with a positivity rate of 30%.

**Figure 6:**
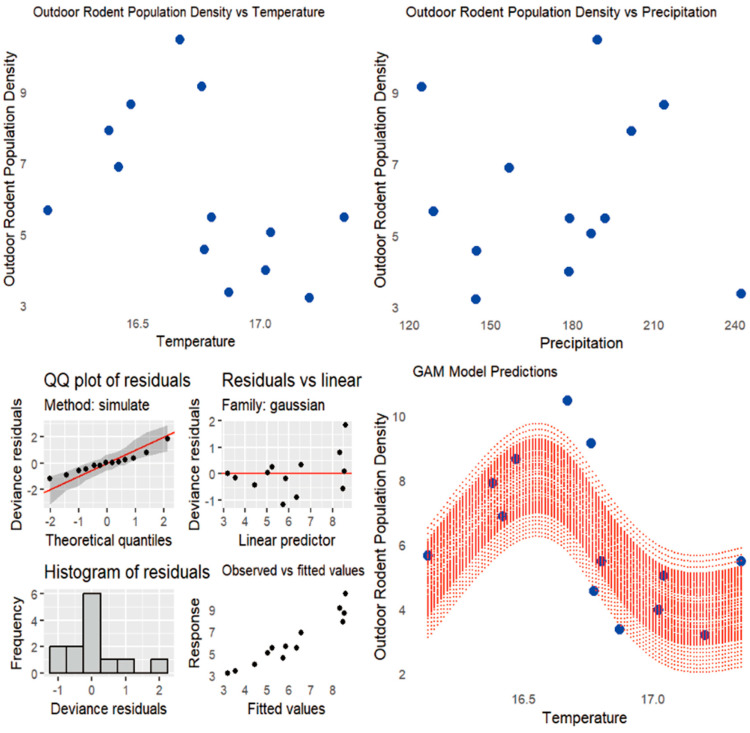
Effects of temperature and precipitation on outdoor rat density in Longyou County from 2011 to 2023 and GAM prediction of future density.

The prediction results of our GAM (Figure 6) indicate that there is a complex nonlinear relationship between the outdoor rodent population density and temperature and precipitation, with the blue points representing actual observed data and the red points representing model-predicted data. Specifically, when the temperature is 16.13 °C and the precipitation is 129.04 mm, the predicted rodent population density is 5.66 units; meanwhile, when the temperature rises to 16.47 °C and the precipitation increases to 213.84 mm, the predicted rodent population density increases to 8.66 units. This indicates that, within this temperature range, the increase in the temperature and precipitation may have facilitated the reproduction and activity of rodents, thereby increasing their density.

However, when the temperature continues to rise above 17 °C, the predicted growth trend of the rodent density begins to slow down and, in some cases, even declines. At a temperature of 17.2 °C and precipitation of 144.48 mm, the predicted rodent population density drops to 3.20 units. This may indicate that high temperatures harm the survival and reproduction of rodents or that, at extreme temperatures, rodents may seek more suitable habitats. Research indicates that the temperature has a significant impact on the reproduction of rodents. For instance, one study [15,16] found that, when the temperature increased from 20 °C to 30 °C, the reproductive success rate of rodents decreased, and the number of offspring was reduced. Furthermore, another study [17,18] pointed out that precipitation also affects rodent reproduction, with wetter years typically leading to higher reproductive rates. These findings suggest that the climatic conditions have a crucial influence on the reproductive potential of rodents and should be taken into account when formulating HFRS prevention and control strategies.

## 4. Discussion

**Figure 7:**
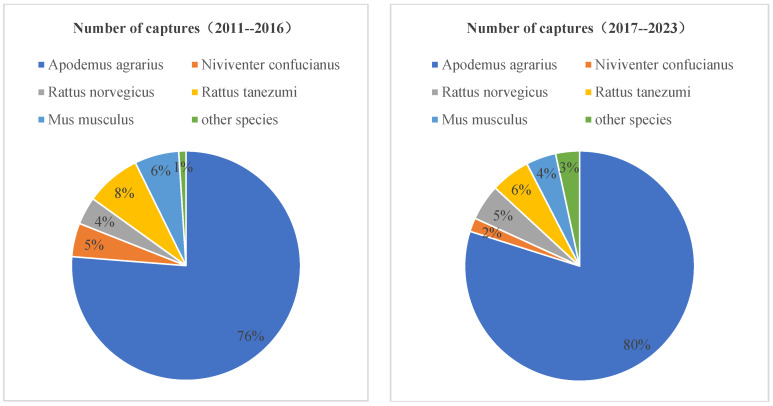
Species of rodents captured in Longyou County, 2011–2023.

The seasonal characteristics of the HFRS epidemic in Longyou County are manifested in two small peaks during the spring–summer and winter seasons [19], consistent with the epidemic trend in Zhejiang Province [20] and also in line with the seasonal epidemic trend of HFRS in China [21,22]. The peak in spring and summer is due to increased contact between people working in the fields and *Apodemus agrarius*, leading to infections, while the winter peak is related to *Rattus norvegicus* entering indoor environments for foraging and breeding [23]. The majority of the population consists of farmers, and the elderly are more susceptible to the illness than the young [24]. This aligns with the characteristics of the mixed epidemic area primarily dominated by *Apodemus agrarius* in the county. The monitoring of host animals in Longyou County from 2011 to 2023 shows that the composition of outdoor rodent species has not changed significantly compared to previous years (Figure 7), with *Apodemus agrarius* being predominant and the host infection rate remaining relatively stable. Indoor rodent species were primarily represented by *Rattus tanezumi*, which marks a significant change from the previous dominance of *Rattus norvegicus*, and the infection rate of *Rattus norvegicus* is notably lower than before. Thus, it is necessary to further strengthen the monitoring of host animals and their virus prevalence.

At the same time, the incidence of HFRS is influenced by various socioeconomic factors [25], including living conditions [26], hygiene practices, and accessibility to healthcare. Research [27] indicates that areas with a higher population density and better education levels tend to have lower rates of HFRS transmission. Furthermore, accessibility to healthcare plays a crucial role in mitigating the impact of HFRS, as timely medical intervention can significantly reduce the mortality and morbidity rates. Studying the spatiotemporal distribution characteristics of HFRS using predictive models [28,29] could enable us to reduce the incidence and mortality rates of this disease in Longyou County. These measures are vital in reducing the incidence of HFRS and alleviating its impact on public health.

The results indicated a significant positive correlation between the outdoor rodent population density and the outdoor rodent virus prevalence, while the temperature and precipitation exhibited a significant nonlinear relationship with the outdoor rodent population density [9,30]. Meteorological factors have a significant impact on HFRS, and this result is consistent with the conclusions drawn in various regions of China [31]. However, this study had certain limitations, such as the GAM not accounting for the effects of confounding factors like air pollution exposure and population mobility. There may also be some interaction between air pollution and the temperature, which could lead to bias in the research findings. Due to limited data, the LR focused on the outdoor rodent population density and the outdoor rodent virus prevalence, without exploring the indoor rodent population density and virus prevalence, which may result in a one-sided outcome.

HFRS mainly affects farmers, particularly those aged 40 to 69. This could be because younger people from rural areas work outside, leaving the middle-aged and elderly to work the fields, raising their infection risks. Despite the 30% positivity rate in antibody tests for those vaccinated in 1995, research shows that the antibody levels drop significantly over time, by 40% after 5–10 years and by over 60% after 10–20 years. With nearly 30 years since vaccination in Longyou County’s key groups, there is an urgent need to boost monitoring, assess the immunization effectiveness, and consider booster shots [32,33] in order to refine the prevention strategies, lower the infection rates, and safeguard at-risk populations’ health.

## 5. Conclusions

This study showed that HFRS in Longyou County remained stable from 2011 to 2023. At the same time, there was a significant correlation between the outdoor rodent density and outdoor virus prevalence, which confirmed that it was a strong predictor of the HFRS transmission risk in Longyou County. These findings highlight the complex interactions between climatic variables (temperature, precipitation), rodent population dynamics, and HFRS incidence, with an increased rodent density associated with increased virulence. The temperature and precipitation further affect rodent reproduction and activity through nonlinear relationships.

In order to achieve the effective control of HFRS, the following comprehensive measures are recommended. First, it is essential to strengthen rodent density monitoring and implement targeted control measures, especially in high-risk areas such as Longnan Mountain. Additionally, outbreak surveillance and interventions should be enhanced, which includes conducting nationwide health campaigns and vaccinating at-risk groups, particularly middle-aged and older people in rural areas. It is also necessary to reassess the immunization coverage in high-risk populations, including individuals who were vaccinated in the 1990s, and provide booster shots as needed. Furthermore, promoting health education and personal protection awareness in rural communities is crucial. Utilizing climate-based forecasting models can help to guide seasonal interventions, and integrating remote sensing, GIS, and mobile Internet technologies to establish a real-time monitoring platform will improve the efficiency of epidemic prediction and response.

## Figures and Tables

**Table 1:** Major host animal species captured in Longyou County, 2011–2023.

Year	Rodent										
	*Apodemus agrarius*	*Niviventer confucianus*	*Rattus norvegicus*	*Rattus tanezumi*	*Mus musculus*	*Maxomys surifer*	*Micromys minutus*	*Rattus nitidus*	*Suncus murinus*	*Microtus fortis*	Total
2011	100	4	3	20	-	4	-	-	3	-	135
2012	88	7	2	1	-	7	-	2	-	-	107
2013	106	8	1	1	-	5	-	1	-	-	122
2014	94	1	5	2	-	1	-	-	-	-	103
2015	93	9	2	3	-	7	-	-	1	-	115
2016	66	5	4	15	-	11	-	-	1	-	102
2017	86	1	3	6	-	5	1	-	1	-	103
2018	92	1	2	2	-	2	-	-	1	-	100
2019	97	-	3	-	-	-	1	-	-	-	101
2020	35	3	18	32	1	5	-	-	6	-	100
2021	114	3	3	8	1	-	-	2	-	-	131
2022	55	8	6	5	-	2	1	14	14	3	108
2023	86	-	3	10	-	-	-	15	8	-	122

## Data Availability

The data presented in this study are available on request from the corresponding author. The data are not publicly available due to privacy.
